# Evolving Digital Health Technologies: Aligning With and Enhancing the National Institute for Health and Care Excellence Evidence Standards Framework

**DOI:** 10.2196/67435

**Published:** 2025-08-22

**Authors:** Shayan Bahadori, Peter Buckle, Tayana Soukup Ascensao, Saira Ghafur, Patrick Kierkegaard

**Affiliations:** 1 Department of Surgery and Cancer, Faculty of Medicine, Imperial College London, London, United Kingdom

**Keywords:** mHealth, mobile app, app, smartphone, digital health, digital technology, digital intervention, wearable device, medical device, machine learning, artificial intelligence, algorithm, model, analytics, implementation, mobile health

## Abstract

The rapid advancement of artificial intelligence (AI)–driven diagnostics and wearable health technologies is transforming health care delivery by enabling real-time health monitoring and early disease detection. These innovations are catalyzing a shift toward personalized medicine, with interventions tailored to individual patient profiles with unprecedented precision. This paper examines the current National Institute for Health and Care Excellence (NICE) evidence standards framework (ESF) for digital health technologies (DHTs) and evaluates the challenges associated with integrating DHTs into existing health and care systems. A comprehensive review of the NICE ESF guidelines was conducted, alongside an evaluation of their applicability to emerging AI and wearable technologies. Key limitations and barriers were identified, with particular focus on the framework’s responsiveness to technologies that evolve through machine learning and real-world data integration. Our findings indicate that while the NICE ESF provides a structured approach for evaluating DHTs, it lacks the adaptability required for rapidly evolving innovations. The framework does not sufficiently incorporate real-world evidence or support continuous learning models, which are critical for the safe and effective deployment of AI-based diagnostics and wearables. To remain effective and relevant, the NICE ESF should transition to a dynamic, adaptive model co-designed with industry stakeholders. By embedding real-world evidence-based strategies and promoting transparency, efficiency, and collaborative innovation, the updated framework would better facilitate the integration of AI-driven diagnostics and wearables into health care systems, ultimately enhancing patient outcomes and optimizing health care delivery.

## Introduction

The swift evolution of artificial intelligence (AI)–driven diagnostics and wearable health technologies is pushing the boundaries of health care delivery, enabling early disease detection, real-time monitoring, and personalized interventions [1,2]. Although these innovations offer significant clinical and operational benefits, they also challenge existing regulatory mechanisms [3,4]. The National Institute for Health and Care Excellence (NICE) evidence standards framework (ESF) [5], designed to assess the value of digital health technologies (DHTs), struggles to accommodate tools that rely on continuous learning algorithms, real-world data, and data interoperability. A pertinent example is AliveCor’s KardiaMobile, an AI-powered wearable electrocardiogram device [6]. This technology highlights several areas where the ESF may fall short, namely, its dynamic learning algorithms, which require continual updates; its dependence on real-world data, which the current framework is not fully equipped to evaluate; and its reliance on data interoperability, which is often hindered by fragmented systems [7]. For instance, despite clinical interest in its potential to support atrial fibrillation diagnosis, the technology faced challenges in aligning with traditional trial methodologies and producing economic evidence acceptable under current NICE criteria [7]. Additionally, concerns around algorithmic bias and the traditionally protracted evaluation timelines further complicate its integration [8]. These characteristics expose a critical misalignment. Although the ESF provides a robust foundation, it is grounded in static, predefined evaluation methodologies that may not sufficiently accommodate the iterative and adaptive nature of modern digital health tools, that is, technologies which are continuously refined and improved through ongoing cycles of data collection, analysis, and algorithmic updates. As such, a transition toward a more agile and evidence-responsive framework is needed to ensure timely, safe, and effective adoption of innovations like KardiaMobile within health care systems.

This paper posits that the NICE ESF must undergo strategic updates to remain relevant and effective in the face of these rapidly advancing technologies. By critically examining the challenges and opportunities presented by AI-based diagnostics and wearable devices, this analysis will propose specific adaptations to the NICE ESF, ensuring that it can support the integration of these technologies into health care systems, ultimately enhancing patient outcomes and optimizing health care efficiency.

This paper presents a viewpoint grounded in existing literature, expert knowledge, and informal stakeholder engagement. No new data were collected for this work. The sources consulted included peer-reviewed articles, policy documents, and gray literature relevant to the topic. Stakeholder perspectives were drawn from prior collaborations, professional experiences, and publicly available materials, rather than formal interviews or surveys.

## The NICE Framework

The NICE developed the ESF for DHTs to provide clear and consistent guidelines for evaluating the efficacy, safety, and economic value of new digital health innovations [5]. The ESF categorizes DHTs into different tiers based on their function and associated risk level, thereby ensuring that the evidence requirements are proportionate to the technology’s impact. This tiered approach ranges from low-risk wellness apps to high-risk digital therapeutics and diagnostic aids [5].

The NICE ESF includes four main components:

1. Evidence for effectiveness, which includes clinical and nonclinical evidence to demonstrate that the technology works as intended and delivers the anticipated health benefits [5].
2. Evidence for economic impact, requiring technologies to show they provide value for money through cost-effectiveness analysis and budget impact assessments [5].
3. Regulatory compliance, data privacy, and security, ensuring that technologies meet regulatory standards and adhere to strict data privacy and security protocols, while also being interoperable with existing health systems [5].
4. Safety and performance standards, ensuring technologies meet safety requirements to protect patients and users, with ongoing monitoring to track performance and adverse events [5].

Although the NICE ESF provides a robust framework for evaluating DHTs, it faces significant challenges in addressing the complexities of rapidly evolving technologies such as AI-based diagnostics, wearable health monitors, and telemedicine platforms. For example, AI algorithms can continuously learn and improve from new data, which necessitates a different approach to evidence generation compared to static digital technologies [9]. Wearables generate real-time health data that can offer insights into patient behavior and health status outside clinical settings, presenting challenges in data integration and analysis [10]. The current NICE ESF, with its reliance on clinical trials and controlled studies, may struggle to adapt swiftly enough to these technological advancements, potentially delaying the evaluation and adoption of cutting-edge digital health innovations.

Furthermore, while this article focuses on the NICE ESF due to its relevance in the UK context, it is worth noting that other international approaches to evaluating DHTs exist. For example, the US Food and Drug Administration’s Digital Health Software Precertification Program [11] emphasizes streamlined review processes for trusted developers, while the European Medicines Agency [12] has explored adaptive pathways to support innovation under conditions of uncertainty. Compared to these models, the NICE ESF offers a detailed stratification of evidence requirements according to risk and functionality, providing a transparent and structured pathway that aligns well with the needs of both developers and decision-makers within the UK health system. However, the existence of multiple international frameworks underscores the importance of regulatory adaptability and cross-jurisdictional learning in digital health innovation.

## The Innovation Ecosystem

The advancement of DHTs is propelled by a complex and dynamic innovation ecosystem, characterized by deep collaboration across academia, industry, government, regulatory bodies, and patient and public involvement [13]. This ecosystem is fundamental to the development and dissemination of transformative technologies, such as AI-driven diagnostics and wearable health devices, which promise to significantly enhance health care delivery and patient outcomes [13].

Central to this ecosystem are start-ups and established companies, which, with substantial backing by venture capital investment and public funding, lead the innovation pipeline [13]. For example, 52North Health Limited [14] recently secured £3.6 million (US $4.8 million) in funding from venture capital firms and the National Institute for Health and Care Research to enhance its innovative cancer patient support platform. This investment enabled 52North Health to expand its personalized medication management solutions, which include tailored reminders, educational content, and real-time health analytics [15]. These investments not only validate the potential of digital health innovations but also highlight the critical importance of interdisciplinary research and development that incorporates the expertise of computer science, engineering, biology, and health care [16].

However, despite the rapid growth of the innovation ecosystem, the NICE ESF remains out of step with the fast pace of technological advancement. Innovators, particularly smaller start-ups, often encounter substantial barriers when attempting to satisfy the stringent evidence requirements mandated by NICE [17]. The traditional emphasis on extensive clinical trials can be prohibitive, especially for technologies that evolve rapidly or rely on continuous data integration and machine learning [17]. Furthermore, without well-established economic evidence, even highly promising technologies may face significant barriers to market entry and long-term adoption [17,18]. Therefore, early engagement with economic modeling and real-world evidence generation, defined as data derived from routine clinical practice outside traditional clinical trials, and technological advancements that inform regular updates to the framework can help innovators prioritize their efforts and improve their chances of achieving reimbursement and scaling effectively [19].

Furthermore, both public and private sectors are increasingly prioritizing high-need, high-cost populations, where DHTs can have the most significant impact [16]. This prioritization amplifies the urgency for a regulatory framework that is both rigorous and flexible, capable of adapting to the unique demands of cutting-edge innovations. To prevent the stifling of innovation, the NICE ESF must evolve to streamline evidence generation processes and foster more collaborative relationships between regulators and innovators. Such adaptations will be essential to accelerating the adoption of promising technologies, ensuring that they reach patients and health care systems without unnecessary delay.

## Challenges and Barriers to Integration

Despite the transformative potential of DHTs, integrating them into the health and care system presents a multifaceted challenge that requires strategic consideration and intervention.

Resistance to change is a major barrier within health care organizations. Clinicians accustomed to established protocols may be hesitant to adopt unfamiliar tools due to concerns over utility, job security, or disruption to workflow [20]. Addressing this requires not only rigorous evidence of clinical value but also engaging providers in co-design and implementation [21]. Structured workshops and pilot programs can help embed these technologies in real-world settings, fostering trust and usability [21].

Cost and infrastructure constraints also impede adoption. Implementing and maintaining advanced technologies can be financially burdensome, particularly in underfunded systems [20,22]. Limited technical infrastructure and expertise, especially in low-resource settings, further restrict integration [23].

Data security and privacy present additional barriers. The sensitive nature of health data demands robust governance frameworks that comply with regulation while maintaining usability. Overly restrictive protocols can hinder clinical workflows, so a balance between security and accessibility is essential [23]. The integration of DHTs into routine care raises significant concerns around the treatment of personal data and patient privacy [18]. These technologies often rely on continuous data collection from wearable sensors and mobile apps, which can include sensitive physiological, behavioral, and location-based information. Ensuring compliance with the UK General Data Protection Regulation and the Data Protection Act 2018 is essential, particularly regarding data minimization, lawful processing, and patient consent [24]. Inadequate safeguards may lead to breaches of trust and reduced uptake among users [24,25]. Privacy-by-design principles must be embedded into the development life cycle of digital tools, incorporating features such as data anonymization, secure encryption protocols, and user-controlled data sharing. In addition, emerging technical solutions like federated learning offer promising pathways to analyze health data without requiring centralized storage, thereby reducing privacy risks [26]. Regulators and developers must collaborate to establish transparent, accountable data governance frameworks that protect individual rights while enabling meaningful innovation.

## Addressing the Problems

To address these barriers, a more dynamic approach to the NICE ESF is essential. Establishing a bidirectional feedback mechanism, where real-world evidence and technological advancements inform regular updates to the framework, is crucial [20,27]. This mechanism would allow for the continuous refinement of the ESF as new data and insights emerge from the use of DHTs in clinical practice. Practical approaches for integrating real-world evidence could include the use of prospective observational studies, pragmatic clinical trials, and the systematic analysis of electronic health records and patient-reported outcomes [27]. Additionally, incorporating methodologies such as federated learning [28] (a technique that enables AI models to be trained across multiple decentralized data sources without sharing raw data) and adaptive trial designs [29] (clinical studies that allow for modifications to trial procedures based on interim results) would enable real-time evaluation of AI algorithms without compromising data privacy. For example, integrating machine learning algorithms into the NICE framework could enable real-time adjustments to evidence standards based on live clinical data, ensuring the framework remains relevant and adaptive [20,30].

Moreover, a supportive policy environment, characterized by government incentives, grants, and favorable regulations, would encourage health organizations to invest in new technologies. Additionally, fostering a culture of innovation within health and care institutions can help overcome resistance [30]. Training programs and continuous professional development can equip staff with the skills and confidence needed to use new technologies effectively [31].

Engaging with health care providers, patients, policy makers, and technology developers through interviews, focus groups, and workshops can provide valuable insights into their needs and concerns [32]. This collaborative approach not only helps in designing user-friendly technologies but also ensures that the solutions are practical and widely accepted [32]. However, a significant challenge with such engagement arises from the conflicting priorities among stakeholders. For instance, technology developers and companies may prioritize the rapid adoption of innovations to gain a competitive market advantage, often pushing for faster regulatory approval or market entry. In contrast, health care providers and regulatory bodies typically emphasize safety, reliability, and long-term efficacy, demanding thorough testing and validation. This tension can lead to conflicts of interest, where the push for speed may compromise the rigor required for ensuring patient safety and data privacy [33].

Regular consultations and iterative feedback sessions with these stakeholders can ensure that the NICE ESF evolves in a way that supports rather than stifles innovation, ultimately enabling the seamless integration of transformative technologies into health care systems.

## Aligning Regulation With Innovation in Digital Health

The integration of DHTs into contemporary health care systems is contingent upon alignment between industry practices and the regulatory standards set forth by the NICE ESF [34]. This serves as a pivotal instrument in ensuring that these technologies are evaluated rigorously for safety, efficacy, and economic value [34]. However, the existing framework poses substantial challenges, particularly for small-to-medium enterprises and start-ups, which often struggle to meet the resource-intensive demands of evidence generation and submission. The disparities in resource availability between established industry giants and emerging innovators exacerbate these challenges, potentially stifling the introduction of groundbreaking technologies that could transform patient care.

Government intervention is critical in addressing these disparities and fostering an environment conducive to innovation. As highlighted in a recent report [35], it is imperative for the National Health Service to embrace digital innovations. Darzi [35] argued that greater investment in digital health care solutions could enhance efficiency, reduce the burden on staff, and improve patient outcomes. He advocated for the expanded use of data and technology to support diagnostics, treatment plans, and remote care. Effective governmental support should extend beyond policy frameworks to include targeted funding initiatives, regulatory flexibility, and infrastructure development aimed at supporting small-to-medium enterprises and start-ups. Hence, the active involvement of key stakeholders, health care providers, patients, technology developers, and policy makers is indispensable. Conducting in-depth interviews, facilitating focus group discussions, and engaging stakeholders through workshops and surveys provide a comprehensive understanding of the barriers and enablers. The government must ensure that these diverse perspectives are systematically incorporated into the regulatory process, thereby fostering a more inclusive and responsive health care innovation ecosystem.

Such strategic engagement with stakeholders should be underpinned by a rigorous methodology, incorporating qualitative research methods such as in-depth interviews, focus groups, and Delphi panels [36]. Analyzing the findings from these activities can elicit rich, nuanced insights into the barriers and enablers of DHT adoption, which can then be synthesized into evidence-based implementation strategies [37]. However, such engagement is not without challenges. Conflicting priorities, such as differing risk tolerances, budget constraints, and timelines between regulators, industry innovators, and clinical stakeholders, can impede consensus-building [36]. To navigate these tensions, structured facilitation techniques, such as modified Delphi processes, stakeholder mapping, and consensus workshops, can be used to ensure all voices are heard while aligning objectives [36]. A relevant example is the National Health Service AI Lab’s Skunkworks program, which uses agile, multistakeholder co-design sprints to identify clinical problems, rapidly prototype solutions, and harmonize feedback from clinicians, patients, and developers [38]. This model demonstrates the value of structured, iterative engagement in achieving cross-sector alignment and fostering adoption readiness. The insights derived from stakeholder engagement should culminate in a detailed report outlining pragmatic strategies for the government to facilitate technology integration [39]. Such strategies should not only identify common challenges but also propose scalable solutions tailored to the unique needs of different health care environments. These insights must then be integrated into a comprehensive, living document that guides government action and supports the continuous evolution of the health care system.

## Recommendations for the Evolution of the NICE ESF

**Figure 1.**
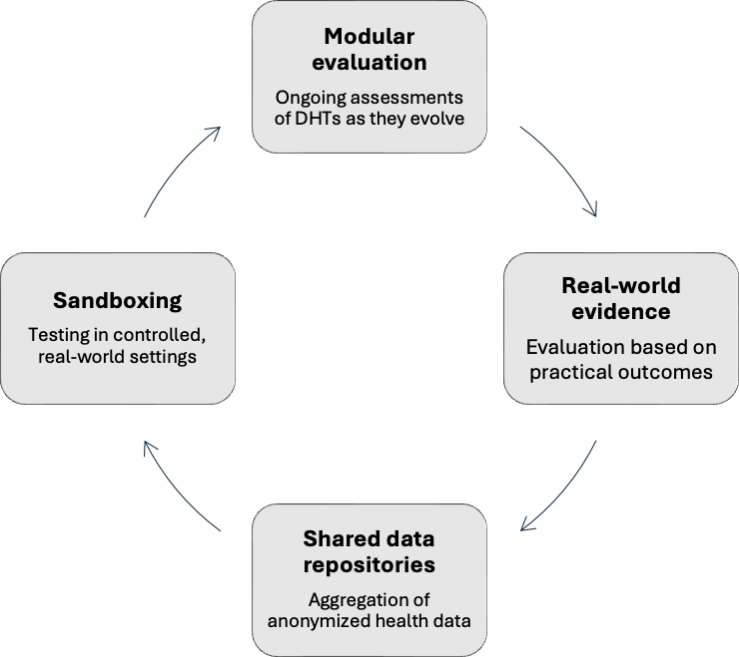
Proposed modification to the NICE ESF. DHT: digital health technology; NICE ESF: National Institute for Health and Care Excellence evidence standards framework.

To address the challenges identified, we propose that the NICE ESF undergo a comprehensive transformation into a dynamic, iterative framework that evolves in real time with advancements in technology (Figure 1). One key innovation is the introduction of modular evaluation processes that would allow the NICE framework to assess different aspects of DHTs as they evolve. For instance, an early-stage prototype of an AI tool or wearable device could undergo an initial review based on preliminary data, while its efficacy and safety are continuously assessed and updated as real-world data accumulate. This iterative approach, supported by ongoing monitoring, would ensure that the NICE ESF remains adaptive to the pace of technological development.

Furthermore, to improve the speed of evaluation and reduce the burden on emerging innovators, we propose the establishment of a “sandboxing” environment within the regulatory framework. This would involve testing DHTs in controlled, real-world settings, where they can be iteratively refined based on real-time feedback from patients, clinicians, and developers. A sandbox model would facilitate rapid experimentation, helping to identify and address potential issues early on while also enabling faster, more flexible approval processes for DHTs. This method has been successfully applied in financial technology sectors (eg, the Financial Conduct Authority’s Regulatory Sandbox), demonstrating its effectiveness in fostering innovation while maintaining consumer protection.

Additionally, shared data repositories should be developed as collaborative platforms where anonymized health data from a range of sources (including wearable devices, health records, and patient surveys) can be aggregated. These repositories would provide developers with access to diverse datasets that can be used to train machine learning models while ensuring compliance with data protection regulations. By pooling data, stakeholders can create more robust AI-driven solutions that are reflective of real-world clinical environments and demographics. This also opens the door for collaborative data governance, where regulators, developers, and clinicians work together to define ethical and security standards for data sharing, ensuring transparency and trust across the health care ecosystem.

Finally, the development of real-world evidence strategies co-designed with industry partners would ensure that technologies are evaluated based on the actual outcomes they produce in practice, rather than just theoretical models or preclinical trials. This collaboration would result in evidence generation that is not only more relevant but also timelier, facilitating the rapid adoption of promising innovations. By integrating such strategies into the NICE ESF, we would enhance the framework’s ability to provide continuous oversight while fostering a more flexible, innovation-friendly regulatory environment.

In conclusion, to facilitate the effective integration of DHTs, the NICE ESF must transition from a static framework to a dynamic, adaptive model. This evolution should include modular evaluation processes, sandboxing environments for real-world testing, shared data repositories, and co-designed real-world evidence strategies. By embedding flexibility and collaboration into the framework, stakeholders can ensure that the NICE ESF continues to support innovation while maintaining rigorous standards for safety and efficacy. These adjustments will not only streamline the adoption process but also enhance patient outcomes, ensuring that health care systems can keep pace with the rapid advancements in digital health technology.

## Abbreviations

AI: artificial intelligence
DHT: digital health technology
ESF: evidence standards framework
NICE: National Institute for Health and Care Excellence
